# Factors impacting the illness trajectory of post-infectious fatigue syndrome: a qualitative study of adults’ experiences

**DOI:** 10.1186/s12889-017-4968-2

**Published:** 2017-12-13

**Authors:** Eva Stormorken, Leonard A. Jason, Marit Kirkevold

**Affiliations:** 1 Department of Nursing Science, Institute of Health and Society, University of Oslo, P.O.B. 1130 Blindern, 0318 Oslo, Norway; 2 Center for Community Research, DePaul University, 990 W. Fullerton Ave., Suite 3100, Chicago, IL 60614 USA

**Keywords:** Chronic fatigue syndrome, Disability, Health care costs, Impacting factors, In-depth interview, Myalgic encephalomyelitis, Patient experiences, Post-viral fatigue syndrome, Trajectory

## Abstract

**Background:**

Post-infectious fatigue syndrome (PIFS), also known as post-viral fatigue syndrome, is a complex condition resulting in physical, cognitive, emotional, neurological, vocational and/or role performance disabilities in varying degrees that changes over time. The needs for health care resources are high, and costly, as is the economic burden on the affected individuals. Many factors may impact the trajectory, and frequently PIFS develops into a chronic condition. Health professionals lack understanding and knowledge, which results in delayed diagnosis, lack of recognition, appropriate treatment, support and practical help. The aim of our study was to explore, from the perspective of persons who had lived with PIFS for four years following an outbreak of *Giardia l*. induced enteritis, factors that may have impacted their illness trajectory and how these factors had played a role during different phases.

**Methods:**

In this retrospective exploratory qualitative study a group of 26 affected adults between 26 and 59 years old were selected for in-depth interviews. A maximum variation sample was recruited from a physician-diagnosed cohort of persons with PIFS enrolled at a tertiary outpatient fatigue clinic. The interviews were audio-recorded, transcribed verbatim and subjected to qualitative content analysis.

**Results:**

Unhelpful and helpful factors were associated with the healthcare system, health professionals and the affected persons were experienced as having an impact on the trajectory. External impacting factors which are related to the health care system, providers and the social security system are misdiagnosis, trivialization of symptoms, unhelpful advice, delayed diagnosis and lack of appropriate help. Internal impacting factors related to the affected individuals were lack of knowledge, overestimating functional capacity, assuming the condition will pass, ignoring body signals and denial. A model of impacting factors in each phase of the trajectory is presented.

**Conclusion:**

Unmet needs may result in unnecessary disability and high societal and personal costs. Enhanced knowledge of impacting factors in each phase of the trajectory may contribute to more timely and tailored health care services and less use of health services. Increased functional capacity, improved health and ability to work or study may reduce the societal costs and the economic burden for the affected individuals.

**Electronic supplementary material:**

The online version of this article (10.1186/s12889-017-4968-2) contains supplementary material, which is available to authorized users.

## Background

An outbreak of *Giardia lamblia*-induced enteritis occurred in Bergen City, Norway, during the summer and fall of 2004 because of contaminated public drinking water [1] subsequently attributed to a leakage in a sewer pipe and an insufficient purification process [2]. Following the acknowledgement of the outbreak, people were instructed to boil the tap water before drinking it and take precautions. The outbreak was acknowledged by the local public health authorities, the National Institute of Health, the Municipality of Bergen City and the Water Work that supplied the water. In addition, the municipality’s insurance company got involved. An evaluation report of the outbreak was issued by the Norwegian Food Administration in 2006 [2]. Some of the persons infected subsequently developed chronic fatigue syndrome [3]. In this study, we explore the experiences of living with this condition 4 years following the outbreak.

People around the world suffer from post-infectious fatigue syndrome (PIFS) [3–5], also termed post-viral fatigue syndrome (PVFS) [6] or myalgic encephalomyelitis (ME) described following outbreaks [7] and used in the international consensus criteria with reference to infectious onsets [8]. No case definitions for PIFS exist. In the case definition used to diagnose the affected persons, the term chronic fatigue syndrome (CFS) is used [9]. However, the term PIFS will be used here because the study sample had a confirmed infectious onset.

Because *Giardia l.* is not endemic in Norway [1], the knowledge among health professionals was low and the parasite not routinely tested for. In Norway, the population is covered by a national health care system funded by taxes. Thus everyone has access to a wide range of health services.

Many developed tiredness or severe fatigue in the aftermath of the infection, making them more or less disabled. Infectious onset is known [8, 10], but the aetiology remains unclear. A loss of 50% or more of pre-illness functional level is required to meet diagnostic criteria, as well as profound fatigue lasting for more than 6 months Fukuda 1994 [11]. Lack of energy and stamina, post-exertional neuroimmune exhaustion (PEM), easy fatigability, sleep abnormalities, immunological dysfunction and neurological and autonomic complaints are prominent features [8]. A range of abnormalities are underlying this condition, among them are decreased cerebral oxygen and blood volume/flow [12, 13], lower body venous pooling [14], impaired delivery of oxygen to muscles [15], delayed restitution period [16], orthostatic intolerance [17] and exercise intolerance [12]. Currently, no curative treatments are available. Thus, affected persons are left with symptom alleviation and having to learn how to manage their symptoms [18]. Complete recovery is rare as less than 5% of individuals recover to pre-illness functional level [19]. PIFS has a greater impact on functional status measured by disease specific norm scores on SF-36 Short Form compared with other diseases such as depression and cancer [20]. Persons with PIFS have lower functional status than persons with HIV [21], rheumatoid arthritis [22], end stage of kidney failure and heart disease [23]. They are also more functionally disabled than persons with multiple sclerosis (MS) [24]. Only terminally ill people with cancer or stroke have functional scores similar to persons with PIFS [23]. Minor exertions, whether physical, cognitive or emotional, may provoke increased fatigue, symptom exacerbation and functional decline [25]. A significant increase in functional capacity is rare [26, 27], thus PIFS results in substantial reduction in quality of life [22]. Globally, millions of people are affected; the prevalence rates are in the range of 0.20%–0.42% [28, 29].

The economic impact on society is substantial [30] and includes loss of employment and productivity that amounts to millions of pounds in the UK [31]. In addition, in the US, more than 18 billion dollars are spent annually for direct health care costs (hospital admissions and out-patient visits, prescription drugs, medical examinations), indirect costs (disability benefits, lost productivity, informal care) [32] and to reduced income tax revenue [33]. Annual national loss of household productivity due to PIFS in the US is greater than in nervous system disorders [34]. The impact on the affected individuals is substantial as well [32, 35], because the personal consequences encompass escalating health care costs, user charges, loss of income for themselves and their household, reduced standard of living, huge out of pocket expenses [32], low educational attainment and lower lifetime earnings [30]. The percentage of participants unemployed at baseline varies between 27%- 65%, and the number returning to work at follow-up ranges from 8 to 52% [19]. The prognosis of returning to work is poor [19]; thus, the reduced functional ability in professional life is substantial [36, 37]. The percentage of individuals living on disability benefits ranges from 25%- 42% [38, 39] and many are dependent on significant others for financial support [40]. Because 90–95% of adults remain chronically ill [19], the multitude and magnitude of the suffering, losses and costs for the affected persons, their families and society, this condition constitutes an important public health issue [41].

To reduce the societal and personal impact, we need not only knowledge of the fatigue experience itself [42], but also what factors impact PIFS in the first years of the illness trajectory. Models may enable health care providers to better understand how PIFS progresses over time and the ill persons’ own reactions to live with it [43]. Models may also help the health care services to deliver improved care, thereby contributing to faster recovery, increased functioning and, hopefully, regaining pre-illness study and work capacity. Few studies have explored trajectory models. Fennell Phase Inventory (FPI), a four-staged model of coping with the condition, comprises life domains such as cultural, physiological, psychological, social and work performance [44]. The model tries to clarify the fluctuating condition and the totality of the illness experience. Ware’s [45] sociosomatic model defines the affected person’s illness as a social experience and is based on the assumption that bodily distress is a result of embodied social problems that are expressed as physical complaints. Whitehead’s [46] trajectory model of three illness-constructed narratives comprises the quest, chaos and restitution phases. These narratives are used to understand the affected person’s illness experience in different phases. However, these models have not explored how the illness and disability trajectory evolves over time during a natural course of the condition following a confirmed infection. Thus, the authors have previously proposed a model of disability trajectory (PIFSDM) consisting of five phases: prodromal, downward, transition, upward and chronic [47]. This model outlines the phases and changes in illness severity and different disabilities over time.

International Classification of Function (ICF) defines disability as “*the outcome or result of a complex relationship between an individual’s health condition and personal factors, and the external factors that represent the circumstances in which the individual lives*” (p.23) [48]. Disability is the lack of or reduced ability to uphold normal financial, vocational and personal standards due to various impairments [48]. The aims of disability management are situational adjustment and behavioural change, but persons with disabilities face barriers in accessing health, social and rehabilitation services [48].

One significant barrier may be a lack of knowledge of how different factors impact each phase of the trajectory. When factors influencing the trajectory have been identified, this knowledge may serve as a tool for assessment and treatment, making it possible to plan and implement tailored treatment programs. This may increase functional ability and optimize health for the affected individuals, reduce barriers and costs to society and improve health care services. To our knowledge there are no studies focusing on the role of impacting factors during a trajectory spanning 4 years. Thus, there is a need for a more complete picture of factors that impact PIFS over time. The aim of our study is to explore, from the perspective of persons with PIFS, factors that affected their illness trajectory and how these factors may have played a role during each phase of the trajectory.

## Methods

### Design

A retrospective exploratory qualitative design was used to elicit the personal experiences of persons with PIFS [49, 50]. In-depth interviews were conducted for data collection [51] and inductive qualitative content analysis was employed to avoid preconceived categories [52].

### Recruitment and description of the sample

According to the Norwegian Prescription Database, about 2500 people fell ill with a gastrointestinal infection caused by the parasite *Giardia lamblia* during the summer and fall of 2004 [1]. The public water supplying half of Bergen City, Norway, had been contaminated by a parasite not endemic in Norway. *Giardia l.* parasites induce enteritis, which causes foul-smelling stools, diarrhoea, weight loss, stomach cramps and bloating [53]. Among those with *Giardia duodenalis* infection many developed post-infectious irritable bowel syndrome (PI-IBS) [54], and a minor group developed severe post-infectious fatigue [3]. Ninety-four individuals suffering from tiredness and severe fatigue were referred to the Neurology Outpatient Clinic at Haukeland University Hospital from August 2005 to September 2007 [3]. A neurologist with long term experience with this condition assessed the referred persons, of whom 58 Caucasians of ethnic Norwegian origin [3] received the diagnosis PIFS according to Fukuda 1994 criteria [9]. This group of 58 persons is termed the ‘total Giardia PIFS cohort’. All in this cohort had laboratory-confirmed *Giardia l*. parasites in their stool during the outbreak. Consent forms, questionnaires and request for participation were mailed from the clinic to the total Giardia PIFS cohort. Background information included functional level [55] [see Additional file 1], signs and symptoms questionnaire [56] [see Additional file 2], socioeconomic and demographic variables and employment/study status. Seventy-six percent (44 persons) of the total Giardia PIFS cohort returned the questionnaires. From this group of respondents, we selected 26 participants for this qualitative interview study. A maximum variation sample [57] was chosen on the basis of differences in background variables such as age, gender, education level, income, marital status, number of symptoms, study/work status and functional disability. Nineteen females and seven males were contacted for an in-depth interview. All of them completed the study. Prior to the *Giardia d.* infection, all participants had been working or studying full time. None had irritable bowel syndrome (IBS) complaints prior to the Giardia infection. Sample characteristics are presented in Table 1.

**Table 1:** Sample characteristics (*N = 26*)

Demographic variables			
Gender			
	19	Females	
	7	Males	
Years of age			
	26–59 (mean 40)		
Education level			
	2	Lower secondary education	≤ 9 years
	4	Upper secondary education	10–13 years
	6	College/university Bachelor	14–16 years
	14	College/university graduate	≥ 17 years
Marital status			
	12	Single	
	9	Married	
	2	Cohabiting	
	3	Divorced	
Work/study status			
	6 4	Females Males	Worked/studied part time & partly dependent on welfare benefits
	13 3	Females Males	Unable to work/study & fully dependent on welfare benefits
Household income^a^			
	4	Very low	
	8	Low	
	9	Average	
	6	High	
	1	Very high	
No. of symptoms^b^			
	14–70 (median 36)		
Post *Giardiasis d*. bowel function			
	6	Experienced no bowel complaints (5 females, 1 male)	
	1	Male missing	
	19	Experienced PI-IBS symptoms (14 females, 5 males)	
	16	Physician confirmed diagnosis of PI-IBS (12 females, 4 males)	

^a^The household income categories were not assigned numerical values and the participants were asked to choose one subjectively

^b^Signs and symptom questionnaire [56]

The participants were retrospectively asked to complete Bell’s Disability Scale [55] [see Additional file 2]. This scale grades the severity of disability on a 10-point grading scale ranging from 100 to 0. 100 = no symptoms with exercise, normal overall activity, able to work or do house/home work full time with no difficulty. 0 = severe symptoms on a continuous basis, bed ridden constantly, unable to care for self.

### Procedure

All of the interviews were conducted by the first author and took place 4 years after the Giardia outbreak. The in-depth interviews lasted one to 2 hours (with a mean of 1.5 h), and the aim was to elicit the participants’ experiences of the four-year long illness trajectory. The opening question was ‘Please, tell me about your illness trajectory and factors that impacted this from the time you fell ill with Giardia infection and until today.’ Prompts were used to further explore their experiences regarding factors that impacted the trajectory, see the interview guide [see Additional file 3].

### Data analysis

As a first step [58], the transcripts were repeatedly read to get an understanding of (a) how the illness/disability trajectory evolved, (b) contextual factors associated with the health care services, the health care providers and the social security system and (c) factors related to the participants themselves that may have impacted the illness trajectory and the participants’ own experiences of it. During this first step we noted a phased trajectory and a variety of potential impacting factors that seemed to be associated with each of the phases. Content analysis is a method of reducing large amount of qualitative data [58], and after reading the full transcripts we used NVivo [59] to extract all material pertaining to the research questions, including the trajectory and impacting factors before we continued the analysis [58]. To avoid pre-conceived categories [52], we undertook a manual inductive analysis of the extracted material as our second step [58]. Meaning units were sentences and passages [60]. Notes and open codes were written in the margins to obtain a preliminary coding frame [58, 60]. During this first open coding cycle [61], freely emerging meaning units were assigned descriptive code labels (Table 2).

**Table 2:** Examples of meaning units, condensed meaning units and codes

Meaning unit	Condensed meaning unit	Code
*I refused to believe it. I denied it, and I know that I almost still deny it, so I kept on working until it no longer was possible.*	Denying being ill had negative consequences	Denial
*I am looking for rehabilitation options. Where can you find this or that?*	Need more knowledge to improve health	Information seeking
*The most important thing is that I accept that I am ill and get to a period where I build myself up again.*	Accepted being ill and taking care of oneself	Acceptance Self-care

The open codes were subjected to categorization during the second coding cycle [52, 61] as coded meaning units were grouped into meaningful categories and sub-categories. Coded data were assigned to mutually exclusive categories. In order to reflect the participants’ experiences, we revised categories and sub-categories several times to identify a meaningful pattern [60]. The main category ‘illness trajectory’ was divided into five subcategories labelled prodromal, downward, turning, upward and chronic. The main category, ‘impacting factors’, was divided into four sub-categories labelled (a) helpful medically-related, (b) unhelpful medically-related, (c) helpful internal and (d) unhelpful internal. An example of how raw data was categorized is found in Table 3. The different phases of the illness trajectory are described in detail elsewhere [47].

**Table 3:** Examples of quotes assigned to illness phases and sub-categories of impacting factors

Main categories: Illness trajectory & Impacting factors					
Category	Illness trajectory				
Sub-categories	Prodromal	Downward	Turning	Upward	Chronic
Category	Impacting factors				
Sub-categories					
Unhelpful medically-related external factors	*Three months untreated*	*The doctor threw me out, wouldn’t have me as her patient.* *An enigma to the GP.*	*Not many [health care providers] have knowledge of this*	*I did not get the [PIFS] diagnosis until March [4 years after the outbreak]*	*Yes, it is difficult to access help, causes very much frustration*
Unhelpful internal factors	*I did not know what it was*	*I have made wrong choices by trying to keep working*	*I did not define myself as sick before I could not get out bed*	*That is obviously what I am still doing [over-estimating my own physical capacity]*	*I see how I go back to the old pattern again when I start feeling better*
Helpful medically-related external factors	*I received a course of antibiotics*		*Yes, when I got diagnosed [by the neurologist] it was actually a relief*		*I think some of the sessions [of the education course] were very helpful*
Helpful internal factors			*[It] just turned when I stared teaming with myself*	*I have become good at saying no and setting limits*	*Now, when I have regained some energy, I will contact [friends] again*

In Table 4, we present an example of codes that were assigned to two of the sub-categories of impacting factors.

**Table 4:** Example of sub-categories and codes

Sub-categories	Unhelpful medically-related external factors	Unhelpful internal factors related to the participants
Codes	• Lacked knowledge • Strained medical encounters • Misdiagnosis • Delayed diagnosis • Trivialized the participants’ symptoms • Attributed symptoms to psychological causes or other conditions • Recommended unhelpful treatments and management advice • Lack of support to the participants • No established system of internal referral at the hospital	• Lacked knowledge • Trivialized own symptoms • Attributed symptoms to a common enteritis that would pass by itself • Attributed symptoms to a nervous stomach • Lack of understanding of having a serious medical condition • Difficulties understanding what was wrong with the body • Tried to fight the condition off by trying to live pre-morbid life • Lack of acceptance • Denial of reality • Lost control over the body • Overestimating own capacity • Unable to reflect on own needs and acquire help as needed

### Trustworthiness

Measures taken to reduce researcher bias and enhance trustworthiness are presented according to the five criteria in the framework of Guba and Lincoln [49, 62]. (1) *Credibility* refers to the extent to which the results are trustworthy enough to be taken into account [49]. A sample larger than usually recommended [57, 63] was used to elicit broad and rich accounts of illness from persons with first-hand experiences [64]. Face-to-face interviews were conducted at a different outpatient clinic at the university hospital rather than the one at which the individuals received treatment. The open and unstructured interview and interactive approach [50] provided the opportunity for the participants to speak freely [65] in their own logic and in their own terms [57] without having any expectations of pre-determined response categories [58]. An interview guide was available to ensure that the participants’ accounts contained experiences in the same areas that pertained to the research questions. The guide included issues identified in previous research and from the first author’s clinical encounters with PIFS. When the few first few interviews were read and interpreted, we noted that the fluctuating illness/disability trajectory and impacting factors showed an iterative pattern. Saturation was reached since no new concepts, impacting factors or pattern emerged after reading and interpreting most of the interviews [64]. The data material amounted to several hundred pages yielding a rich source for understanding what factors impacted the trajectory [49].

(2) *Dependability* refers to how reliable the findings are [49]. To establish rapport, trust and facilitate the dialogues, the interviews were conducted in a conversation-like manner [50]. The interviewer strove to have an open mind, to be an attentive listener and to avoid too much interference [50]. The accuracy of data was secured in different ways: (a) unfamiliar dialect words or idioms were asked to be explained or elaborated: (b) unclear statements were rephrased and the participants asked to clarify: (c) the interviews were audiotaped and transcribed verbatim and (d) the audio-recordings and transcripts were checked for consistency by the first author [50]. To extract data pertaining to the trajectory and impacting factors we used *NVivo* software [59], which made it easy to create memos and annotations and to track back to raw data. Field notes, methodological, analytical and ethical reflections were recorded in a reflective journal the same day as the interview took place [50]. The reflexive journal [66] also kept track of the study regarding considerations, decisions made and the rationale behind them. The first author’s perceptions regarding the culture and context surrounding the diagnosis, the topic and persons with this condition were reflected upon and written down to reveal the preconceptions before participant enrolment [67]. The preconceptions, either from personal experience, discipline-based or from clinical work, that emerged from this endeavour were acknowledged, reflected upon throughout the study and recorded in the reflexive journal [66]. Self-critical and analytical reflections regarding researcher skills, potential role conflicts and the risk of losing the analytic researcher positioning were recorded and acted upon in the research team.

(3) *Confirmability* refers to the accuracy of data and interpretation [49]. The first and third author read the interviews and manually coded the extracted text independently. In order to secure coding consistency, interpretive disagreements of meaning units, codes, categories and sub-categories were discussed until agreement was reached [65]. A draft of the result section was read by the third author who endorsed the interpretation and findings. All three researchers agreed on the final findings and how to interpret the information provided by the participants. The first author is a registered nurse who has gained extensive personal experience and has more than a decade of professional experience with persons affected by this condition by working as a long time lecturer for group-based education courses at different hospitals in another health region in the country. The second author is an established and experienced researcher in the field, whereas the third author has extensive research experience in other medical conditions and some experience with research on post-infectious fatigue syndrome.

To check findings from the in-depth interviews, we used method triangulation [49]. In addition to in-depth interviews, we used Bell’s Disability Scale [see Additional file 1] to ensure confirmability regarding the evolvement of the trajectory. The participants’ own illness accounts corroborated their self-rated functional level at different points in time. To demonstrate the link between raw data, interpretation and content of each subcategory, multiple quotes from various participants are included in the result section [65, 68].

(4) *Transferability* refers to the question of whether the findings in our study may be transferred to other contexts [49]. A detailed description of design, sample, recruitment, data collection, analysis and context/culture is provided [65]. In addition, to help judge whether our findings would be applicable in other contexts, groups or points in time, our detailed presentation of the findings may be helpful. It is, however, the reader’s own decision to make judgements about the transferability of our findings [69].

(5) *Authenticity* refers to the issue of whether our findings represent the authentic realities experienced by our participants [62]. Multiple voices and different experiences of functional ability levels and impacting factors in all phases of the trajectory are presented. To demonstrate that multiple realities are presented and captured in our understanding and interpretation of the explored phenomenon, we have included quotes from several participants in each category.

### Ethical considerations

All parts of this study adhered to the principles of the Declaration of Helsinki [70]. Approvals are stated in the Declarations section. To avoid compromising their identity, each participant was assigned an ID number, and all information regarding identity was removed from the data material. The persons included consented in writing to voluntary participate in our study. Since only selected persons from the total Giardia PIFS cohort could participate in this qualitative study, the selecting procedure was described in the request letter. Every participant was informed in the letter about the study’s purpose and the right to withdraw at any time without consequences and this was repeated orally before commencement of the interview.

Interviewing vulnerable persons may trigger painful emotional reactions. When crying spells occurred in a few participants, they were asked if they wanted to terminate the interview. However, they orally renewed their consent to continue [71] as they wished to complete their story of living with the condition. Persons with PIFS may experience symptom flare-ups following any kind of exertion, have a low capacity limit and can become easily fatigued [42]. To reduce harm and minimize any inconvenience the interview room was dimly lit and quiet, and the interviewees sat in a recliner with foot stool and were offered light refreshments during the interview.

## Results

The participants experienced a five-phased illness trajectory: prodromal, downward, turning, upward and chronic phase [47]. Here we present impacting factors during the illness trajectory, organized into external/internal and helpful/unhelpful impacting factors for each phase, as experienced and described by the participants. External factors are associated with the health care system, the social security system, health care providers and society, whereas internal impacting factors are related to the participants themselves.

### Impacting factors associated with the prodromal phase

#### Unhelpful medically-related external factors

The participants blamed the Municipality of Bergen City for becoming severely ill:

I was infected by Giardia and have received the diagnosis [post-infectious] ME… caused by Bergen Municipality [because of insufficient purified public drinking water] (P6).

Because *Giardia duodenalis* is uncommon in Norway, and only a few cases of imported cases occur yearly [1], health care providers were inexperienced and lacked knowledge. Many participants felt that their general practitioners (GPs) trivialized their symptoms. The following is a sample statement: ‘“*I think I’ve got Giardia.” [The GP:] “No! Absolutely not! You haven’t.” Like [my complaints] was a typical female [thing]*’ (P19). When full health was not regained as expected, the GPs related their symptoms to stress, psychological problems or other causes: *‘I went to see the GP, and he assumed I suffered from eating disorder, which I of course didn’t do*’ (P8). Lack of knowledge among GPs and their tendency to not take the symptoms seriously caused a prolonged time to reach diagnosis. Participants waited as long as ‘*Three months untreated’* (P2). This caused unnecessary long-lasting enteritis that may have contributed to higher symptom burden and a decline in functional ability.

Individuals with *Giardia l*. infection had been followed up by the Department of Medicine, as many of them suffered from tiredness and irritable bowel syndrome. A few with suspected PIFS were referred by a gastroenterologist to the Neurology Outpatient Clinic. However, there seemed to be a lack of a well-functioning referral system between the hospital departments:

Yes. I’ve got this irritable bowel. They found this out [at Dep. of Med.]. No, it’s really through the media that I found out that there were several others that struggled with the fatigue. No, [none at the hospital told me about referral to the Outpatient Neurology Clinic]. I even asked the medical staff at the Department of Medicine if [the fatigue] could be another problem. It took quite a few years before I was referred to a neurologist because of the fatigue (P4).

A few GPs made a referral to the clinic after being pushed by the ill persons themselves: ‘*I asked my GP to refer me [to the fatigue outpatient clinic] because I had heard about the neurologist*’ (P21). Some participants referred themselves by contacting the clinic, whereas others needed help from family members to do it. One participant stated: ‘*My father called the neurologist’* (P19).

Some participants feared they were seriously ill and needed urgent medical care, but they no longer had faith in their GPs. The GPs lack of knowledge and failure to take their clients seriously created an atmosphere of distrust and a strained medical encounter, which only added to the participants’ emotional burden:

I was afraid that there was some... vital organs began to fail or... I felt extremely sick. So I said, “Just forget the emergency room and forget my doctor. Just call directly to the hospital. Call the neurological department and get me admitted. And just forget my GP, because now we have tried so much with her.” Actually I had mentioned [the neurologist] to my GP earlier as I knew there was a person who worked with [fatigued persons], but the GP replied: “You will not gain access there. It’s no use in trying to be examined by him.” Imagine how it was to receive such a message from your own GP! The neurologist phoned me after a few days and had booked an appointment… a tremendous relief (P19).

#### Unhelpful internal factors

At first, the gastrointestinal problems and tiredness were experienced by the participants as symptoms of a common infection that occurs among individuals from time to time. The participants did not understand what was wrong with them, and some of them also related it to stress. They made statements like: ‘*I didn’t know what it was… thought it was a nervous stomach… but I didn’t feel stressed*’ (P2), or *‘[I] thought it was other factors*’ (P16). As the participants thought the infection would pass by itself, they pushed themselves to continue working or studying, with a negative result as they experienced a deterioration of their health, expressed by one participant as: ‘*What I’ve done… hasn’t been wise*’ (P3).

### Impacting factors associated with the downward phase

External treatment-related factors, external societal moral expectations and internal impacting factors seemed be associated with the downward phase.

#### Unhelpful treatment-related external factors

Seemingly, the GPs did not listen attentively to catch the difference between fatigue and depression or understand the participants’ complaints. Thus, several GPs confused the profound fatigue with depression, eating disorder, burnout, or psychological problems. Misdiagnosis in some cases resulted in unhelpful treatment with antidepressants that made some participants worse. One participant said, ‘The GP told me: “*You’re depressed... called hidden depression*.” She prescribed antidepressants to me that made me extremely much worse’ (P19).

As mentioned above, many participants experienced emotionally strained relationships with their GPs: ‘*The doctor threw me out, wouldn’t have me as her patient: “I’m asking you to find another doctor”*. *And I’d seen her for three years*’ (P10).

Some participants or their caregivers came to realize the diagnosis through newspapers or a television program before meeting with their GPs. Diagnostic delay of PIFS seemed unhelpful: ‘*I should have known what was wrong with me at an earlier stage. I would have made other choices. This probably made me worse’* (P26).

As GPs seemed to lack knowledge of PIFS, it made it difficult for them to handle the situation appropriately. PIFS was *‘An enigma to the GP*’ (P16). Commonly, GPs recommended more physical activity. This proved unhelpful, as the participants experienced that an increase in activity only resulted in increased disability and symptom flare-up: ‘*It was completely wrong [to exercise], but my doctor was so fiercely determined about it,’ one participant said. The GP forced me to go for walks… [and I] ended in bed for one week with terrible pain*’ (P19).

Because of lack of appropriate medical advice, the participants tried to do their best on their own. As they were not in control of their condition, they did not know when to stop doing things that made them worse, and this also seemed to contribute to the downward trajectory. The participants were afraid that the way they had managed their condition would harm them ‘*for the rest of [their] lives*’ (P10).

For shorter or longer periods, some participants experienced being in need of help, but the lack of practical and/or financial help from the public health care system, the social security system (Norwegian Labour and Welfare Organization) or the Municipality’s insurance company may have contributed to symptom flare-up, relapses and further functional decline as the participants felt they did not have enough opportunities to rest and restore their energy level:

[If I had received help] maybe I didn’t have to push myself and had avoided the tough decline (P7); I had a desperate need for rest (P24); I’ve made wrong choices by trying to maintain work. I’ve had no choice [because of poor economy] (P10); If I had been given child care assistance at once, this wouldn’t have taken so long (P11).

#### Unhelpful external societal moral expectations

Twenty-four of the 26 participants did not experience embarrassment by being ill with PIFS: They said, ‘*No, no. no, it’s not embarrassing, it’s frustrating*’ (P25). However, many of the participants experienced embarrassment and humiliation connected with their disabilities such as cognitive impairment, easy fatigability and lack of energy and stamina when failing to meet other people’s socially grounded expectations:

I have such a fear that… when amongst other people, if someone would ask me about something, I can’t provide an answer. Periodically I was like that at home too. It was problematic for me to walk to the mailbox. Imagine if I meet someone who talks to me… who wants to ask me questions (P7); I had to excuse myself all the time because my abilities did not suffice (p10).

The invisibility of PIFS was experienced as embarrassing and emotionally draining as some participants thought other people looked at them as lazy persons who did not want to contribute to society:

Yes… it’s so invisible, apparently I look normal. You feel that people may think that you might be a little bit… listless, a little unenterprising… I’ve had that feeling, especially in the beginning (P24).

The participants wanted to live up to the societal moral values and expectations of being hard-working, reliable colleagues and productive individuals. However, complying with these social values seemed unattainable at this stage. They pushed themselves at the expense of their own health and were drained of energy to the extent that they could not spend time with their family or work:

I don’t think it’s the right thing to be on sick leave… it’s about keeping one’s honour intact. You don’t want to overload your colleagues. I tried to [endure at work], but had to take more sick leave. I spent all [my strength at work]; nothing was left for [the spouse] or the children (P24).

#### Unhelpful internal factors

The participants did not realize that they were suffering from a serious medical condition and continued to manage their lives with PIFS in the same way they had in their prior healthy life. Working or studying as in pre-illness life made them more and more fatigued and increasingly functionally disabled. They did not understand why they continued to deteriorate: ‘*I don’t understand it myself*’ (P16).

The fatigue and fatigability in PIFS was experienced as something else than tiredness in ordinary life: ‘*[T]here’s a huge difference between being tired and fatigued*’ (P26). However, it took a long time before they realized that the fatigue and concomitant symptoms were signs of a medical condition: ‘*I didn’t define myself as sick before I couldn’t go out of bed*’ (P8).

Although the participants were cured of their *Giardia d*. infection, they continued to feel that something was wrong with their bodies, as they experienced numerous unpredictable fluctuating symptoms that were difficult to understand. All the participants were overwhelmed, experienced a sense of chaos and were unable to comprehend what was going on:

I’ve hardly grasped this, no logic… symptoms… It’s not like you have this today and that tomorrow, or you have all at once, because it alternates. It can be everything in one day or it can be something else the next day. And the severity varies considerably, and it can vary within a day, and it can vary within a week… and it’s related to activity… and it does not help to think positively, that’s not enough. It hasn’t been in a way that I could say that this is cause or effect… not been in a way that I can predict anything (P9).

During the first years, the participants tried hard to live as they had in their healthy lives: ‘*I fought and fought… tried insanely hard [to live my old life]*’ (P16). They thought more physical activity was a solution for their increasing fatigue: ‘*I started exercising... maybe that was the solution... if you exercise, you get more energy*’ (P4), they thought, but this did not work. Some expressed that they had put their life on hold and just were waiting to wake up one morning as healthy so they could go on with their former life. Some said: ‘*I have in a way put [my life] on hold*’ (P13). Denial and lack of acceptance seemed to delay improvement:

I refused to believe it. I denied it and I know that I almost still deny it. So I kept on working until it no longer was possible… [only] lying on the couch and in bed (P8); I’ve never accepted being sick (P20).

The self governed the body in a fight to regain the pre-illness life and pushed the body to perform as expected when healthy:

Before [I got ill] I knew exactly what the body could provide. [Then, when I got this] I lost my autonomy [and control] over the [body’s capacity to perform on demand] (P1); [I]t’s gone beyond what I can control, no influence on it (P16).

As the participants had not learned how much their ill body could provide in terms of energy to perform as expected, they overestimated their capacity limit, both at work, when studying or in their personal life, resulting in crashes and reduced functional ability:

I hadn’t learned to stop in time… years passed with crash after crash (P26); Yes, I pushed myself so hard that I ended up in hospital (P16).

The participants sought to find different explanations for their suffering: ‘*I tried to find reasonable explanations based on [earlier] experiences*’ (P23). Since they lacked knowledge, they used their ‘old’ experiences and coping strategies to deal with their new challenges.

During the downward phase, the participants had still not learned by trial and error that increases in symptom severity and worsening of fatigue were warning signals of the body’s capacity limit. Thus the body’s physical, cognitive and emotional signals were ignored, ‘*There were clear signs, easy to overlook’* (P1).

The participants seemed to lack or have a reduced cognitive capacity and ability to reflect on their own needs for help or assistance because of fatigue, energy loss and cognitive impairment:

I haven’t really reflected on my needs… don’t even know what the health care system can offer (P20); I was so ill that I didn’t think… failed to reflect (P7).

### Impacting factors associated with the turning phase

#### Unhelpful external factors

The time to reach the PIFS diagnosis ranged from four months to four years (median one year and seven months), and the participants received little or no appropriate information from health professionals prior to receiving their PIFS diagnosis:

Not many [health care providers] have knowledge of this. Never heard anything about [post-infectious] ME, that this has been a distinct diagnosis (P17); [The] diagnosis I didn’t receive before March [2008] (P20).

#### Helpful external factors

When the diagnosis was provided, it was possible for the participants to get some explanation and understanding of their condition. From the neurologist, they received medical advice on how to deal with PIFS:

[The] pieces came together. It felt good to receive [the diagnosis and] guidelines telling you that you should actually take it easy, listen to your body and include rest periods and do things gradually… doesn’t help to push it. I really needed that message (P9); Yes, when I got diagnosed it was actually a relief (P4).

#### Helpful internal factors

During the turning phase, the participants realized that the relationship between body and self had to change, and that they had to listen to what their body signals were trying to tell them, ‘*I haven’t listened to them, probably the reason for the situation I’m in now*’ (P20). The time had come to let the body take charge. When the body took control, they experienced that they were able to find out how much their body could perform without provoking symptom flare-ups or hampering improvement:

If I pushed a little too hard… very unwise. Yes, [my desires and driving forces have been much stronger than the body’s capacity], that’s how I see it. I’ve probably pushed my body too much all the time (P13).

In this phase the participants realized they could no longer keep up with their pre-illness lifestyle, but had to change how they perceived their condition. This engendered a process of recognition and acceptance: ‘*I’ve had to really go into myself. I’ve realized that I’m sick. I’ve started thinking like that. Pretending to be healthy, when you are not, is not working*’ (P13).

The first years without control over their own bodies and lives posed a great challenge, but, during the turning phase, the participants began working with themselves to regain control:

I looked at how I could work with myself as a project. I see new avenues. The most important is that I accept to be in a phase where I build myself up again (P3); I’ve more control. Now I’ve to do this, now I’ve to that (P11).

The participants realized that lifestyle changes were necessary, including taking time off, resting more, changing their focus from others’ needs to their own, being laid back and finding a harmony between the body and self. In other words, they started to care for themselves:

[It] just suddenly turned when I started teaming with myself (P3); In retrospect I see that… recharge… is an important key factor. I daren’t think of the consequences if I had just continued as I did (P16).

### Impacting factors associated with the upward phase

#### Unhelpful internal factors

When improvement occurred and the energy level increased the participants experienced getting better and wanted to do more. As everyone had a strong wish to regain normalcy − that is, their pre-illness lifestyle − the participants continued to overestimate their capacity. When they overexerted themselves they experienced relapses, increased disability and symptom flare-ups that lasted for days, weeks or months. The upward phase was characterised by a pattern of improvement and setbacks:

That’s obviously what I’m doing [overestimating my own physical capacity]. I see the fact that I do it in everyday life too, because I feel very much better. Yeah [easy to overdo], especially now, it’s very easy to overdo (PT13).

#### Helpful internal factors

The participants regained some control through trial and error learning which made them realize that pushing themselves beyond their body’s limit drained them of their energy and made them worse. Thus they gradually became better at setting limits:

I’ve become good at saying no and setting limits... don’t wear myself out to satisfy other people’s need (P18); I’ve to be very rigorous, stingy regarding what I spend the energy on (P26).

After years of trial and error, the participants started noticing the body’s warning signals in order to find out its capacity to avoid or minimize relapses in everyday life:

[By] listening to the body and making the right choices and prioritizations I may come up to a level that I can be satisfied with, it’s a key to continue to get better than I’m today (P16).

The participants had realized during the turning phase that the way they had handled the situation during the downward trajectory was inappropriate. When they received the PIFS diagnosis, they actively started to seek information on how to handle their condition. They looked for information on the internet, in magazines, at support associations’ websites and elsewhere to obtain knowledge about PIFS and how to manage it:

It had been useful with some advice along the way: This is wrong. This you mustn’t do. You should stop. This is right (P10); [I’m] looking for rehabilitation options. Where can you find this or that? It’s a little harder to treat one-self. Self-treatment isn’t always as easy. It’s nice to have someone who can support (P2).

During the turning phase they had realized that their personal, professional and social activity level prior to the *Giardia l*. infection had become counterproductive because it increased their symptom burden and resulted in a severe decline in their abilities to function in all life domains. Thus, in the upward phase they began to modify their lifestyles and develop self-management strategies. Their focus of attention had shifted from the outer world − that is, on satisfying other people’s needs and complying with moral societal expectations − towards themselves and their own needs. They worked at coming to terms with being ill and determining how they could take care of themselves in order to regain health. Since the participants wanted to get better, they had to focus on themselves. This required them to lower the demands they put on themselves and let their own needs take priority over societal moral values:

At my sister’s I gave this message: “Enough is enough! I’ve to lie down on the couch.” I begin to learn that I’ve to pay a little more attention to my body (P20); We can tolerate that it does not necessarily have to be perfectly clean (P18).

Planning ahead, prioritizing, having pre-emptive rest, rest breaks, and post-exertional rests contributed to fewer incidents of crossing the capacity limit, more predictability and subsequent increased functional level for most of the participants:

[I] take a nap in the afternoon before I’m going to meet some friends to endure a bit longer (P20); I’ve had to prioritize as tough as nails, become more critical of what I spend efforts and time on and whom I spend time with (P9).

The participants experienced that by making changes in their daily lives, adapting to their situations and channelling their energy in a more conscious way prevented stressful situations and facilitated a better balance between rest and activity:

That helped me… pacing… self-management... energy conservation (P2); [I’m] better at resting. I improve faster (P21).

The participants experienced that acquiring help and taking actions to lower their energy expenditure, including home help or child care assistance, moving to a more easily maintained living facility or more quiet surroundings and withdrawal from energy draining commitments, facilitated the opportunity to rest more. Therefore, they experienced an improvement in their functional level:

I had much more help… a great improvement (P24); I [moved] to an apartment with all facilities… close to the shops (P23).

All the participants were forced to reduce their activity levels to facilitate improvement, but how much depended on how severely affected they were. Those who were working realized they worked too much and either reduced working hours, changed to less strenuous jobs or stopped working. The students either reduced hours of studying per day, read far less than before, switched to more easy study subjects, skipped exams, and/or took leave or dropped out:

50%... a temporary job, much easier, less physically demanding than my ordinary job (P13); [I] study only four hours… read much less, switched to more easy study subjects (P16).

### Impacting factors associated with the chronic phase

#### Unhelpful external factors

Many participants still experienced lack of support from the health care system and the social security system to obtain welfare benefits, or they struggled with poor economy and to get compensation from Bergen Municipality’s insurance company, ‘*I can’t trust them*’ (P2). A common refrain was, ‘*Yes, it’s difficult to access help… requires very hard work… causes very much frustration’* (P2). Poor economy and the fight to obtain benefits was a very energy draining emotional burden that seemed to hamper improvement.

#### Helpful external factors

The participants had been followed up by an interdisciplinary team at the Neurology Outpatient Clinic after being diagnosed with PIFS, and the GPs had learned more along the way. In the fall of 2007 the hospital organized an educational course that was delivered in five sessions for the cohort of persons with PIFS. For various reasons, not everyone attended, but many found that learning from other persons in the in the PIFS cohort was helpful with regard to mastering their own challenges and learning how they could improve their own health:

What I learned most from was meeting with other people in my situation so that I could talk to them [and exchange experiences] (P6); I think some of the sessions were very helpful (P26).

#### Unhelpful internal factors

The loss of their prior lifestyle and functional ability provoked common reactive psychological worries. In addition, they still found it challenging to find their activity limit, thus there was still a risk for overdoing, even after four years:

I do too much, at work too, because of [an economic] need. I see how I go back to the old pattern again when I start feeling better, that’s probably very risky (P13).

Lack of energy and stamina, easy fatigability and high symptom burden made the participants feel older than they actually were. This coupled with a strained economic situation, was experienced as scary and emotionally draining:

I feel old prematurely... live like a loner, like an old lady. I feel like an octogenarian in a forty years old body. Everything hurts, stiff, weak… before I was very physically fit and climbed on walls and ran upstairs and carried things. That’s over. I’m so scared when I go from here… everything hurts… you are thinking, living like you are supposed to do when you are in your eighties. I feel like I’m drained. I feel like my body has gone through a huge process of... as if my body has been inside of a dishwasher for several years, or inside a dryer, [and] that my body has been thrown around, and nerves have been on edge (P10).

Many still suffered from IBS complaints. Some symptoms had abated, whilst others experienced the same symptom burden, or had become worse again:

The recent weeks have been the best in a long time (P4); I’ve become weaker and weaker than I’ve been the last years, much more fatigued… like a zombie at home (P29); Stomach pain, diarrhoea and sweating all day… the body trembles, headache and the stomach growls, flatulence (P20).

The experiences of being severely incapacitated, having a poor economy and lacking financial support from the social security system or insurance company made participants force themselves to work to provide for themselves and their offspring. This made their everyday very emotionally challenging, drained them of energy and seemed to be counterproductive in regard to improvement of health:

I have no social life… a limited quality of life, to say the least. What should I do? What is right? But I have not ... I can stop working, I might lie down, but I have no one that puts bread on the table for me and my daughter the next day [if I don’t have income]. What do I do then? Will the Child Protection Services take my daughter away? (P10).

The participants trying to maintain full-time work needed longer and longer sick leaves. Those who tried hardest to keep going exhausted the body more and more and eventually dropped completely out of work or studies. Others were able to work or study part time, less than 50%.

I’m on disability benefits (P20); Now it’s fifty-fifty for me when I’m working 50%… 50% welfare benefits (P26); I’m much better… at school six hours every day, max (P16).

How much the participants had improved their functional level during the natural course of four years varied a great deal as a few had hardly or only slightly improved whilst some had improved markedly, and a few had experienced a new decline. However, none of the participants had regained pre-illness health or functional level.

Despite a high symptom burden, the participants were not pre-occupied with attributing symptoms to a physical cause as they already were aware that their symptoms emerged in the wake of an objectively confirmed infectious disease.

#### Helpful internal factors

With proper diagnosis, education and years of experiential trial and error learning, the participants were more competent and confident in managing their daily lives. Listening to their bodies and adapting to their needs increased their functional capacity. With increased energy levels, the intolerances to sensory stimuli abated to varying degrees, and the participants were able to more or less take part in social life, see friends and enjoy cultural events, with a few exceptions: ‘*The social life with concerts and theatre, and cinema and lots of people, I’ve had to put on hold. I feel a bit like I’m heading back now, and that’s very good*’ (P14).

Most participants had improved their cognitive abilities, but this had not happened to everyone. As one individual said, ‘*I have no memory. What happened last week, it’s gone*’ (17).

Despite being ill for four years, being more or less unable to function and having a limited quality of life, the participants had an optimistic view of their future, wanted to get better, and had a strong wish to become productive individuals and optimize their potential for a higher functional level or becoming healthy again: ‘*I hope I'll still get better*’ (P16). However, some had no social life, and many were uncertain about the future as they were aware of the risk of relapses:

I’ve no social life [today]… Life will never be as before. I might have done something wrong and will be damaged for the rest of my life, and the quality of life will be limited, but I want to maintain hope (P10).

The self and the body were more in balance, as the participants had improved their abilities to set limits and use self-management strategies: ‘*I take very small steps at a time. [I’ve] been burned so many times that I’m not betting more than that*’ (PT17).

### The participants’ retrospectively self-rated ability to function

**Fig. 1:**
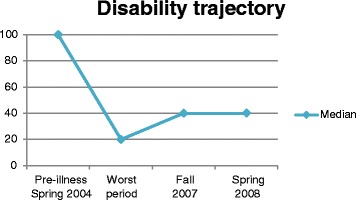
Median sample disability scores measured by Bell’s Disability Scale (*N* = 26)

**Fig. 2:**
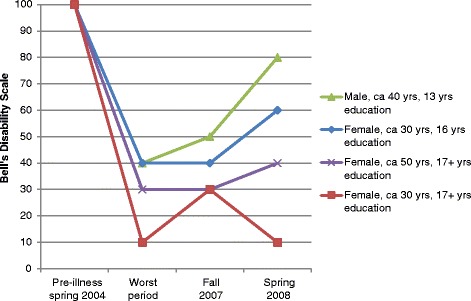
Example of four different disability trajectories

As mentioned previously, the participants scored their functioning retrospectively by Bell’s Disability Scale before becoming ill, at nadir and twice during the upward phase. The downward trend comprises both the prodromal phase and the downward phase of PIFS, i.e. from being healthy in the spring of 2004 until the nadir of the disability trajectory. The transition between the phases was not distinct but overlapped. In this qualitative study, our intention is not to present correlational statistics but to present descriptive statistics of median sample scores (Figure 1) and examples of individual scores (Figure 2) as complimentary means of visualizing the trajectory. In both figures, the downward trend comprises both the prodromal and downward phases. The point in time, when the ability to function in daily life reached its nadir, differed among the participants. Because the nadir occurred sometime between 2004 and 2007, it cannot be specified in the figures.

Pre-illness, the spring of 2004, the median sample score was 100, and this is in agreement with background data. The nadir median sample score was 20. This reflected moderate to severe symptoms at rest, inability to perform strenuous activity, expected overall activity levels of 30–50%, inability to leave the house except rarely, bed confinement for most of the day and inability to concentrate for more than one hour a day. The median sample score in the fall of 2007 and prior to the interviews in 2008 was 40, which reflected moderate symptoms at rest, moderate to severe symptoms with exercise activity, overall expected activity level of 50–70%, no home confinement, inability to perform strenuous duties, ability to perform light duty/desk work 3–4 h a day and rest period requirements.

The participants’ own scores displayed individual disability trajectories. To visualize differences in trajectories, and therefore different disability levels at different phases, the trajectories of the most and least severely disabled participants plus two in between, are presented (Figure 2).

If the participants experienced relapse, they regressed for a while. Some participants hardly improved their functional level, whereas others improved but later experienced a new decline in ability to function. The participants’ accounts revealed many factors that they felt impacted their illness course and fluctuating disability level, either unhelpful or helpful. None of the participants regained pre-illness functional level.

## Discussion

The aim of our study was to explore experiential factors impacting each phase of a four-year long illness trajectory. Lay people who fall ill with a new and serious condition such as PIFS, lack knowledge and skills to manage their new condition. Thus, they need to rely on the health care system for adequate help to prevent or limit the consequences and be provided with proper advice and treatment to facilitate improvement in health and functional ability. However, our findings suggest that when the participants struggled hardest to cope with increasing disabilities during the downward phase, they received unhelpful or counterproductive advice from health care providers and lack of medical and financial support. This combination may have contributed to the severe downward trajectory that the participants experienced.

As early diagnosis is assumed to improve prognosis [72], a delayed diagnosis and treatment of *Giardia l.* enteritis [1] and a delayed PIFS diagnosis and education about the condition kept the participants ill and untreated for a prolonged time with potential harmful consequences, such as avoidable severe decline in health and ability to function. Other external unhelpful impacting factors identified in our study have also been found previously. These include strained medical encounters [73], misdiagnosis [74], GPs uncomfortable making a diagnosis [75], experience of disbelief, lack of knowledge and medical support and psychologizing of symptoms [73], unsupportive GPs [46, 76], being let down by the health professions [77] and poor communication with health care providers [78]. Our findings of context-specific experiences are in line with previous qualitative studies [79].

During the downward phase, the participants were overwhelmed by their situation and lacked the ability to reflect on their own needs [42]. Thus, it took them a long time to accept being seriously ill and recognize that keeping up with their pre-illness lifestyles was counterproductive and that they had not handled their situations appropriately. Their needs for help, whether practical, financial or educational, had not been taken seriously, nor adequately provided for. Because they had no prior experience with PIFS and lacked support from their GPs and a medical framework provided by the diagnosis, the participants were unable to interpret their illness experience and cope with it in a health promoting way. Instead, they interpreted their new challenges through the lens of being healthy. Using coping strategies from pre-illness life showed to be maladaptive during this phase of the trajectory [80]. Our findings of internal unhelpful impacting factors are consistent with previous studies; they include being in denial [81], or overwhelmed [77], feeling insecure [82], trying to fight the condition off [77], feeling guided by moral values [83, 84] and disruption of the self and the body [84], reacting emotionally [81], returning too early to work and taking insufficient time to recuperate [83]. The negative impact on the downward trajectory was doubled as unhelpful internal factors and maladaptive coping strategies [80] only added to the unhelpful external factors.

During the turning phase, the participants started to accept that they were ill and recognized the importance of adapting to their needs. With the interpretive framework grounded in the diagnosis, helpful advice, practical help, medical and financial support and education, the participants were able to manage their new life in a more adaptive way. Acquiring knowledge is important to regain control [85] and reduce feelings of chaos and insecurity [46]. In line with our findings, important internal factors during the improvement phase were keeping the energy expenditure lower than the perceived energy at any given day [86], setting limits and planning activities [46], pacing to avoid symptom exacerbation [87], getting sufficient rest, monitoring activities, making lifestyle adjustments, occupational shift, reduction in social life [88], and adaptation to the fluctuating symptoms [89]. Consistent with previous findings, the participants in our study were more confident in managing their condition and its limitations in the chronic phase and used more adaptive coping strategies [90].

Demographic characteristics such age, gender and education in our study are consistent with previous samples [1, 87, 91] and did not seem to have a significant impact. A similar pattern of impacting factors was identified during the phases of the illness trajectory in all of the interviews. However, the ability to function varied among them, and some were more affected by impacting factors than others.

Acceptance is crucial [77], as is access to practical aids, home help, adapting the housing situations [77], gaining insight into PIFS [77] and having a sense of control [77] to facilitate improvement in functional ability. However, contextual elements such as lack of proper health services, lack of referral system and time- and energy-consuming difficulties with the social security system and the municipality’s insurance company imposed a significant emotional burden on these severely ill persons, draining them of energy that could be used in a more health-promoting way. A strained economic situation due to the inability to work, difficulties obtaining benefits and economic support were unhelpful and hampered improvement. Without financial means, some individuals were forced to work or to work too much, and were unable to pay for home help, childcare assistance, physiotherapy or psychological services needed to help them deal with their reactive psychological challenges. Other contextual elements were lack of knowledge among health care providers and no established system for taking care of persons with PIFS.

Unhelpful internal impacting factors played an important role, but these impacting factors could have been significantly reduced if an appropriate health care system, medical care and support systems had been in place. Helpful external impacting factors came too late and were insufficient, resulting in a prolonged period during which unhelpful internal impacting factors played a significant role. Impacting factors played an important part during the four-year long illness duration, but it is impossible to know to what extent the experienced impacting factors influenced the symptom severity and the disability level in each phase, as symptom severity and disability trajectory are influenced by underlying pathological mechanisms in various body systems [11].

### Models as tool for assessment and treatment planning

In this study we propose a new model of external and internal factors that either impact positively or negatively in each phase of the illness/disability trajectory. This model may enhance the understanding of the fluctuating trajectory and make it possible to identify which phase a person with PIFS is located in, to assess factors that impact the person and to tailor the treatment in accordance with impacting factors. Our model may function as a robust lens to understand how the illness and the impacting factors change over time as the affected persons learn to live with their chronic condition. In example, there is empirical evidence for phases of coping identified by Fennel’s Phase Inventory [92, 93]. As well as dynamic changes in coping, there are also dynamic changes in our proposed model of impacting factors.

Fennel’s [44] coping model revealed negative internal impacting factors such as denial, the fight to continue with pre-illness lifestyle and external negative factors such as misinterpretation of symptoms, trivialization of symptoms, stigma and societal moral values. Whitehead’s [46] model of constructed illness phases identifies one positive impacting external factor: receiving diagnosis is important to reduce the emotional burden. Negative external factors were unsupportive GPs, the struggle to obtain a diagnosis and the contested view of the condition. Positive factors were information-seeking to find out how to improve health, changing lifestyle, setting limits and adjusting workload. In Ware’s [45] model of social illness course negative impacting factors included poor economic situations, moral work ethics, stigma and positive factors included shifting to easier jobs, working less hours during the week and setting limits and prioritizing tasks.

Contrary to the findings by Fennel’s [44], Ware’s [45] and others [94], the feeling of stigma attached to the diagnosis of PIFS only played a small part in this study. However, several participants experienced embarrassment due to various disabilities. The reason for this may be that the participants were part of a healthy group that had fallen ill by drinking contaminated public water, an emergent public health issue, thus they were not to be blamed for the reason to contract the *Giardia l*. infection.

Although the aforementioned models have reported some negative and positive impacting factors found in our model, the impacting factors have not been organized into positive and negative external and internal factors in a structural framework. In our framework we present impacting factors that are associated with different phases of the trajectory. We also present findings of retrospective self-rated trajectory by using Bell’s Disability Scale [55], and these findings of ability to function at different points in time confirm the participants’ accounts of their illness course and help to visualize the fluctuating character. This scale may assist in monitoring the trajectory. Our previously proposed disability model of PIFS (PIFSDM) [47], the models of Fennel [44], Ware [45], Whitehead [46] and the proposed model of impacting factors in PIFS (PIFSIM) during a four-year long trajectory, presented here, may be seen as complementary means to understand the evolving condition and facilitate clinical observations and help planning treatment in accordance with the needs of each phase. All models are flexible and take into account the relapsing and remitting nature of the condition. Persons with PIFS need respect and empathy from health professionals and help to rebuild their lives [95], in addition to improved services and support [96].

### Implications for practice, education and research

Most health care providers in clinical practice have little or no knowledge of PIFS and do not feel confident in making the diagnosis or deciding how to treat persons with this condition. Enhanced knowledge among professionals who encounter this group may increase awareness of the seriousness of PIFS, the fact that it may follow various infections, how the trajectory unfolds and impacting factors associated with different phases. A better understanding of the condition may contribute to more accurate assessments of symptoms and functional ability and facilitate planning and organizing of appropriate health services. Individually tailored treatments and support services may be more health-promoting, effective and less costly. Educated health care providers would likely provide better quality of care in order to enhance the functional levels of affected individuals and reduce the use of health care resources. Lack of knowledge suggests a need for inclusion of PIFS (or post-infectious ME) in the curriculum at all levels of health care education. The proposed model of impacting factors has to be interpreted with caution, however. Our findings need to be replicated in other populations with this condition. Future research might be directed at evaluating what interventions are most appropriate in each phase of this condition to find out which ones will have the most helpful effect on reducing the downward trend, facilitating the improvement process and increasing the ability to function.

### Strengths and limitations

The strengths of our study are open-ended interviews of persons with first-hand experiences, a well-defined maximum variation sample and a confirmed infectious onset. We do not draw any conclusions about causality between the *Giardia l*. infection and PIFS because this is outside the scope of a qualitative interview study. Besides, the pathophysiological process from contracting an infection to development of PIFS is still unknown. Our interview sample is selected from a defined cohort, the total Giardia PIFS cohort. The participants had been provided with the PIFS diagnosis by an experienced neurologist at Haukeland University Hospital, Norway, prior to recruiting the sample and conducting the interviews. In addition, the outbreak constituted a public health issue, acknowledged by public health authorities and the Municipality of Bergen City. Our study has several limitations. Aspects associated with a number of impacting factors may have been left out. Several participants reported memory gaps, and as our study is retrospective, our findings are therefore vulnerable to recall bias. None were bedridden or housebound at enrolment, but had been during the time of their lowest functional level. As our sample was recruited from a tertiary clinic, the findings may not necessarily reflect experiences of population-based samples. Our sample had an infectious onset, giardia duodenalis, and may deviate from samples wherein PIFS was contracted following exposure to other infectious agents. The findings may not be applicable to all affected persons or other populations with gradual onset without a confirmed infection because this condition is considered heterogeneous. However, our findings may be transferable to other populations of post-infectious syndrome because many of our findings are consistent with previous research.

## Conclusion

The participants experienced several impacting factors, whether external, internal, helpful or unhelpful, associated with each of the five illness phases. The participants expressed several unhelpful internal factors that impacted the downward phases, and these made them make unhelpful or potential harmful decisions on how to manage their illness. The downward phases are critical periods of time, and the combination of unhelpful internal impacting factors coupled with the unhelpful external impacting factors related to the health and social care system, the social security system (Norwegian Labour and Welfare Service) and the municipality’s insurance company during this time, may have had a negative impact, resulting in an unnecessary severe disability and prolonged time of being severely disabled. Helpful internal factors such as acceptance and years of trial and error learning contributed to a turning phase and made the participants take more control during the upward phases. Poor economy was experienced as a negative impacting factor hampering improvement. Both practical and financial support was experienced as critical factors, because this made it possible for the affected persons to rest enough to regain energy and increase their functional abilities.

The health care system, including the specialist and GP services, and the social security system, had more or less failed, despite the fact that internal and external impacting factors during the illness trajectory have been known for many years. The participants’ experiences of unhelpful professional attitudes, in addition to lack of knowledge and support, suggest a significant potential for improvement regarding care, treatment, education and assistance. There is a need for better cooperation between individuals with PIFS, the health care providers and the social security system. If more appropriate health care services and other support systems had been in place earlier, adapted to each illness phase, the illness trajectory and severity may have had a more favourable outcome. Insufficient or faulty health care services may pose a burden on both the individual and society, in terms of severe disability, lost productivity and inefficient and unnecessary costly health care services.

Enhanced knowledge may change health care providers’ attitudes to persons with PIFS in a positive direction and contribute to improved and flexible health and social services, and other support systems. Our presented model of impacting factors associated with a five-phased trajectory of PIFS may provide a tool to develop more tailor-made health care and other services during the different phases that ultimately may lower costs to the individual and society.

## Additional files

Additional file 1 Bell’s Disability Scale. (PDF 65 kb)

Additional file 2 Signs and symptoms questionnaire. (PDF 31 kb)

Additional file 3 The interview guide. (PDF 35 kb)

## Abbreviations

CFS: Chronic fatigue syndrome
FPI: Fennel Phase Inventory
Giardia d: Giardia duodenalis
*Giardia l*: *Giardia lamblia*
GP: General practitioner
IBS: Irritable bowel syndrome
ICF: International classification of function
ME: Myalgic encephalomyelitis
PEM: Post-exertional malaise
PIFS: Post-infectious fatigue syndrome
PIFSDM: Post-infectious fatigue syndrome disability model
PIFSMIF: Post-infectious model of impacting factors
PI-IBS: Post-infectious irritable bowel syndrome
PVFS: Post-viral fatigue syndrome
