# Supplementary material for: Factors impacting the illness trajectory of post-infectious fatigue syndrome: a qualitative study of adults’ experiences
Source: BMC Public Health. 2017 Dec 13;17:952. doi: 10.1186/s12889-017-4968-2 (PMC5729235; doi:10.1186/s12889-017-4968-2)
Supplement: Supplementary file 3 — The interview guide. (PDF 35 kb) [file 12889_2017_4968_MOESM3_ESM.pdf]

Additional file 3: The Interview Guide

Factors impacting the illness trajectory of post-infectious fatigue syndrome

The aim of our interview guide was to assist in exploring factors impacting the course of the illness from being healthy to becoming ill with *Giardia lamblia* enteritis that subsequently developed into post-infectious fatigue syndrome that had lasted for four years. In-depth interviews follow their own logic. Thus, the questions/themes addressed by the questions and probing questions may occur spontaneously and not in the sequential order listed below.

Various factors may influence the course of an illness. Factors may be associated with you as a person or may be associated with external factors not relating to you. Factors that influence the course of an illness may be helpful or unhelpful.

- From your experience, what helpful and unhelpful factors influenced the illness course related to the Giardia infection?
- Can you provide examples of both helpful and unhelpful factors?
- What factors do you think were associated with you?
- What factors do you consider were associated with external factors?

2. What factors, both helpful and unhelpful, do you consider influenced your illness during the time when the development of post-infectious fatigue syndrome and you became more and more ill?

- Can you describe factors, events or incidents that you think influenced the illness when your health declined?
- Can you provide examples of both helpful and unhelpful factors?
- What factors do you think were associated with you?
- What factors do you consider were associated with external factors?

3. From your experience, what factors do you assume influenced your illness at the time you were most severely ill?

- Can you provide examples of both helpful and unhelpful factors?
- What factors do you think were associated with you?
- What factors do you consider were associated with external factors?

4. At some point in time your health may have started to improve. What factors do you think influenced the illness course at that time?

- Can you describe both helpful and unhelpful factors?
- What factors do you think were associated with you?
- What factors do you consider were associated with external factors?

5. If you look back at the four years that have passed, have you had any relapses? If yes, what factors do you assume may have influenced this?

- What factors do you think were associated with you?
- What factors do you consider were associated with external factors?

6. If you look back at the last year, what factors do you consider having influenced your illness?

- Can you describe both helpful and unhelpful factors?
- What factors do you think were associated with you?
- What factors do you consider were associated with external factors?
